# Fire-mediated dieback and compositional cascade in an Amazonian forest

**DOI:** 10.1098/rstb.2007.0013

**Published:** 2008-02-11

**Authors:** Jos Barlow, Carlos A Peres

**Affiliations:** 1 Department of Biological Sciences, Lancaster Environment Centre, Lancaster UniversityLancaster LA1 4YQ, UK; 2 Museu Paraense Emílio Goeldi (MPEG)Avenida Magalhães Barata 376, Belém, Pará 66040-170, Brazil; 3 Centre for Ecology, Evolution and Conservation, School of Environmental Sciences, University of East AngliaNorwich NR4 7TJ, UK

**Keywords:** savannization, tropical forests, tree mortality, resilience, climate change

## Abstract

The only fully coupled land–atmosphere global climate model predicts a widespread dieback of Amazonian forest cover through reduced precipitation. Although these predictions are controversial, the structural and compositional resilience of Amazonian forests may also have been overestimated, as current vegetation models fail to consider the potential role of fire in the degradation of forest ecosystems. We examine forest structure and composition in the Arapiuns River basin in the central Brazilian Amazon, evaluating post-fire forest recovery and the consequences of recurrent fires for the patterns of dominance of tree species. We surveyed tree plots in unburned and once-burned forests examined 1, 3 and 9 years after an unprecedented fire event, in twice-burned forests examined 3 and 9 years after fire and in thrice-burned forests examined 5 years after the most recent fire event. The number of trees recorded in unburned primary forest control plots was stable over time. However, in both once- and twice-burned forest plots, there was a marked recruitment into the 10–20 cm diameter at breast height tree size classes between 3 and 9 years post-fire. Considering tree assemblage composition 9 years after the first fire contact, we observed (i) a clear pattern of community turnover among small trees and the most abundant shrubs and saplings, and (ii) that species that were common in any of the four burn treatments (unburned, once-, twice- and thrice-burned) were often rare or entirely absent in other burn treatments. We conclude that episodic wildfires can lead to drastic changes in forest structure and composition, with cascading shifts in forest composition following each additional fire event. Finally, we use these results to evaluate the validity of the savannization paradigm.

## 1. Introduction

The only fully coupled land–atmosphere global climate model that is currently available predicts a large-scale and substantial reduction in precipitation over the Amazon Basin during the twenty-first century, leading to a widespread dieback of forest vegetation (Betts *et al*. 2004; Cox *et al*. 2004). These catastrophic predictions are highly controversial, not least because the thresholds for drought-mediated dieback remain highly uncertain, and are currently based upon a single digital vegetation model (TRIFFID) that is not consistent with the long-term historical stability of core Amazonian forests (Haberle & Maslin 1999; Sternberg 2001) and may underestimate forest resilience to drought (e.g. Nepstad *et al*. 2007; Saleska *et al*. 2007).

Conversely, the structural and compositional resilience of Amazonian forests may have been overestimated, as the vegetation models fail to consider the potential role of fire in the degradation of forest ecosystems (Betts *et al*. 2004). Fire plays a significant role in tropical ecosystems and is a major evolutionary force shaping the structure and composition of forests worldwide (Bond & Keeley 2005), and the geographical extent of many tropical rainforests is limited by fire as well as edaphic and climatic factors (Bowman 2000; Hoffmann & Moreira 2002; Russell-Smith *et al*. 2004). Furthermore, most tropical rainforest trees are poorly adapted to fire stress, and even low-intensity forest wildfires can lead to the mortality of more than 40% of stems 10 cm and above in diameter at breast height (DBH; see Barlow & Peres (2006*a*) for a review of studies).

Many of the ongoing and predicted changes in the Amazonian climate increase the risk of fires spreading into forests. For example, climate change is likely to be accompanied by increased air temperature and dry season length over large regions of the Amazon forest (Cox *et al*. 2004), and a probable increased frequency of severe seasonal droughts initiated by El Niño–Southern Oscillation (ENSO) events and Atlantic sea surface temperature (SST) anomalies (Li *et al*. 2006). These severe supra-annual droughts are known to both augment and further desiccate the available fuel load, forcing forests over the flammability threshold (Nepstad *et al*. 2004; Ray *et al*. 2005) with potentially disastrous consequences. An estimated 20 Mha of tropical forest in South America and Southeast Asia succumbed to drought-induced fires as a consequence of the 1997–1998 ENSO event (see Barbosa & Fearnside 1999; Cochrane *et al*. 1999; Siegert *et al*. 2001; Page *et al*. 2002; Cochrane 2003; Alencar *et al*. 2006), and the extreme 2005 drought—triggered primarily by elevated middle Atlantic SST anomalies—led to a rapid proliferation of fires in normally fire-resistant parts of southwestern Amazonia (Aragão *et al*. 2008).

Many of the ongoing changes in anthropogenic activity either facilitate or directly increase the spread of fire (Cardoso *et al*. 2003). For example, large-scale forest clearance leads to reduced precipitation (Oyama & Nobre 2003), and edge creation and selective logging increase forest flammability (Uhl & Buschbacher 1985; Nepstad *et al*. 1999; Cochrane & Laurance 2002; Alencar *et al*. 2006). Moreover, many deforested areas are subsequently replaced by secondary forests (e.g. Lucas *et al*. 2000), which can become rapidly desiccated and flammable during dry periods (Ray *et al*. 2005). Finally, the increased physical access across previously undisturbed parts of the Amazon through mega-development projects (e.g. Peres 2001) encourages the immigration and spread of human populations dominated by small-scale farmers who routinely use fires to clear agricultural plots (e.g. Nepstad *et al*. 2001; Cardoso *et al*. 2003). This frontier expansion increases the susceptibility of forests to fires and provides the ignition sources that are responsible for initiating most fires.

The aim of this paper is to assess the potential fate of the Amazon forest under drier climatic conditions by examining the long-term consequences of contemporary wildfires. We evaluate post-fire forest recovery and the consequences of recurrent fires for the patterns of dominance of tree species by surveying tree plots in unburned and once-burned forests examined 1, 3 and 9 years after fire, in twice-burned forests examined 3 and 9 years after fire and in thrice-burned forests examined 5 years after the most recent fire event. Specifically, we evaluate the post-fire forest recovery trajectories in terms of stem density, and the consequences of recurrent fires for the abundance of the dominant tree species. Finally, we use this novel dataset to re-evaluate the potential outcome of a widespread fire-mediated vegetation dieback in terms of a largely irreversible phase shift in Amazonian forest ecosystems.

## 2. Material and methods

This study took place in the Rio Maró basin of westernmost Pará, central Brazilian Amazonia (2°44′ S, 55°41′ W). This region is dominated by dense lowland non-flooded (*terra firme*) forests, but includes small enclaves of edaphic savannahs (*campinarana*) on white-sand soils, and narrow portions of seasonally flooded forests (*igapó*) along the Rio Maró. The forest plots inventoried in this study are classified as yellow latosols of medium texture (according to the Brazilian soil classification system; EMBRAPA 1981), and have a high (but variable) sand fraction and a relatively low moisture retention capacity (J. Barlow & C. Peres 1998, unpublished data). The climate is characterized by a rainfall of 2041 mm yr^−1^ (range 1287–2538 mm yr^−1^; 1992–1997) and a strongly demarcated dry season lasting three to five months (see Barlow & Peres 2006*b*).

The modern history of forest wildfires in this region dates from the early 1990s, when fires escaped from ‘roçados’ (small agricultural clearings) into the surrounding forest. Arguably, the most important fire events in the twentieth century occurred between October and December 1997, at the end of the longest dry season in living memory. These fires affected ∼1100 km^2^ of logged and unlogged primary forests, previously burned forests (providing our twice-burned treatment) and secondary forests in the Rio Arapiuns–Maró basin (Peres 1999; Nelson 2001). Additional severe dry seasons (especially 2001–2002) have led to recent wildfires in the last decade (providing our thrice-burned treatment), but these fires were less extensive and more patchy than those of late 1997.

In 2007, we recensused twelve 0.25 ha forest plots (10×250 m) that had been examined at least once previously, including four plots located in unburned forests, four in once-burned forests, two in twice-burned forests and two in thrice-burned forests. The unburned and once-burned plots were established in 1998–1999, one year after the 1997–1998 fires, and were resampled both 3 and 9 years after these fires (in 2001–2002 and 2007). The twice-burned forest plots first burned in the early 1990s, and were first sampled 3 years after the second fire (in 2001–2002) and resampled 9 years after the second fire (in 2007). The thrice-burned forest plots were sampled in 2007, 5 years after they had burned for the third time. Where our previously georeferenced rectangular plots were no longer obvious (i.e. in twice-burned forests), the transect points marking the beginning and the end of each plot were relocated using a global positioning system and its original compass bearing.

We recorded all trees and lianas 10 cm and above in DBH in each plot, but excluded those stems with more than half of their basal trunk outside the plot. Diameter measurements were taken at breast height (approx. 1.3 m), or immediately above the tallest buttress whenever this exceeded 1.3 m. During the 2007 field campaign, measured stems were identified to the level of family, genus or species by J.B., based on personal knowledge, local names provided by a highly experienced field assistant, and following Gentry (1993) and Ribeiro *et al*. (1999). At the same time, we also identified all common species of shrubs and saplings above 1 m in height and below 10 cm in DBH in twelve 1×50 m subplots, with four subplots placed in each unburned and burned forest treatment. We did not count bamboo stems or other non-woody vegetation, although their abundance varied considerably in different burned forests (figure 2).

Voucher specimens were not collected, and we restrict our analysis of species composition to the most abundant stems (defined as stems with a density greater than 10 trees ha^−1^), using a coarse-scale level of identification to emphasize the broad patterns of compositional change and minimize error. As we are primarily interested in evaluating patterns of regeneration responses in terms of the abundance of stems resulting from the most recent fire event, our examination of changes in composition (table 1) focuses on the smaller stem classes (10–20 cm in DBH) and saplings below 10 cm in DBH. This analysis of a limited number of plots is designed to provide a preliminary evaluation of patterns of change, and present working hypotheses that can be tested by studies of long-term forest dynamics based on marked trees, and by wider-scale and well-replicated examinations of post-fire regeneration across the Amazon Basin.

## 3. Results

### (a) Changes in stem density

**Figure 1:**
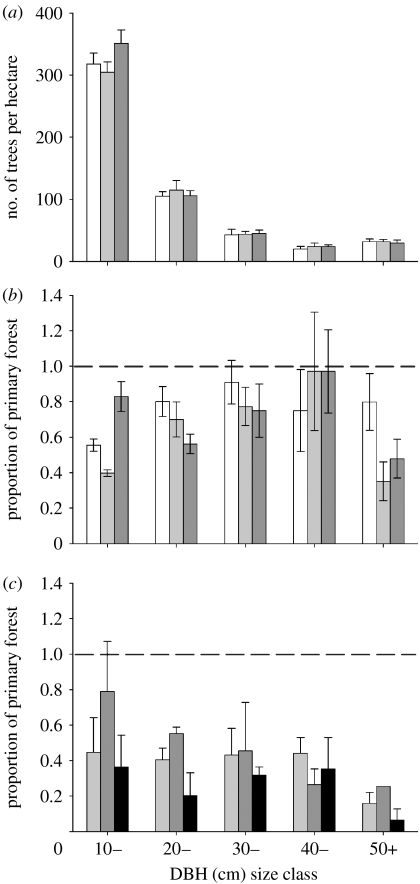
(*a*) The number of trees per hectare across five different size classes in unburned primary forests sampled in 1998–1999 (open bars), 2000–2001 (light grey bars) and 2007 (dark grey bars). (*b*) Average tree density proportional to the level recorded in unburned forests in once-burned forests sampled in 1998–1999 (years since most recent fire: open bars, 1), 2000–2001 (light grey bars, 3) and 2007 (dark grey bars, 9). (*c*) Average tree density proportional to the level recorded in primary forests in twice-burned forests sampled in 2000–2001 (light grey bars), twice-burned forests sampled in 2007 (dark grey bars) and thrice-burned forests sampled in 2007 (black bars, 5). The dashed horizontal lines represent tree density in unburned primary forest.

The stem density recorded in unburned primary forest control sites was stable over time, and there was no significant change in the total number of trees recorded or in any of the five individual size classes (figure 1*a*; Kruskall–Wallis tests, *p*>0.6 for all comparisons within size classes). We therefore compared the number of trees in each size class in burned forest plots with the average number (across years) of trees recorded in unburned primary forest plots for the same size class.

In once-burned forests, there was a decline in the abundance of live trees in both the smallest (10–20 cm DBH) and largest (50 cm and above DBH) size classes between 1 and 3 years after fire (figure 1*b*; see also Barlow *et al*. (2003*b*), which include data from three additional plots that had since been clear-cut and could not be sampled in 2007). There was strong recruitment into the 10–20 cm DBH size classes between 3 and 9 years post-fire, and the abundance of these smaller stems was approaching the numbers recorded in primary forests. There was little evidence of any significant change in the abundance of stems in the larger size classes (20 cm and above in DBH) between 3 and 9 years after the fires, although the variance among replicate plots was high (figure 1*b*).

Within twice-burned forests, there was a marked recruitment of stems into the 10–20 cm DBH size class between 3 and 9 years post-fire. There was a slight increase in stems 20–30 cm in DBH over the same period, but no discernible trends were observed in other size classes (figure 1*c*). Thrice-burned plots that burned for the last time in 2001–2002 had fewer trees across most size classes than twice-burned forests, but this was especially noticeable in the smaller (10–30 cm DBH) and larger (50 cm and above DBH) size classes.

### (b) Changes in stem composition

There was a clear pattern of tree community turnover among the most common stems of small trees in the 10–20 cm DBH size class. For example, the dominant morphospecies in unburned primary forests were always substantially less abundant in once-burned forests, and very scarce, if not entirely absent, in twice- and thrice-burned forests (table 1). Once-burned forest plots were dominated by long-lived pioneers, such as *Cecropia* spp. and *Jacaranda copaia*, which were not recorded in unburned primary forests and were either much less abundant or absent from twice- and thrice-burned forests (table 1). Twice-burned forests were dominated by *Pseudobombax* sp., which was absent from unburned and once-burned forests, and thrice-burned forests were dominated by *Cordia* sp., which were only infrequently recorded in other burn classes.

**Figure 2:**
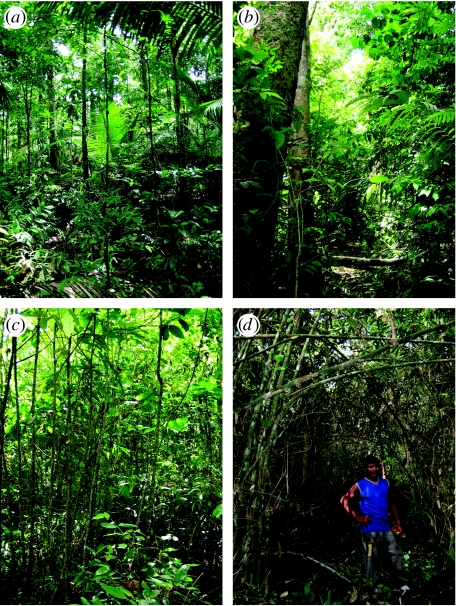
Understorey regeneration characterizing (*a*) intact primary forests, (*b*) once-burned forests 9 years post-fire, (*c*) twice-burned forests 9 years post-fire and (*d*) thrice-burned forests 5 years post-fire. Photos were taken in the Arapiuns–Maró river basin in January 2007 by J.B.

A similar pattern of plant community turnover was evident with the shrubs and saplings (above 1 m tall and below 10 cm in DBH), many of which reached reproductive maturity in the understorey. The most abundant species recorded in once-burned forests (*Palicourea guianensis*) was rarely recorded elsewhere, and the most abundant species in twice-burned forests (*Aparisthmium cordatum*) was much less abundant in once- and thrice-burned forests. Finally, the most abundant species in thrice-burned forests (*Cordia* sp.) was rarely encountered elsewhere in any of the undisturbed or other fire-disturbed treatments (table 1; figure 2).

## 4. Discussion

**Figure 3:**
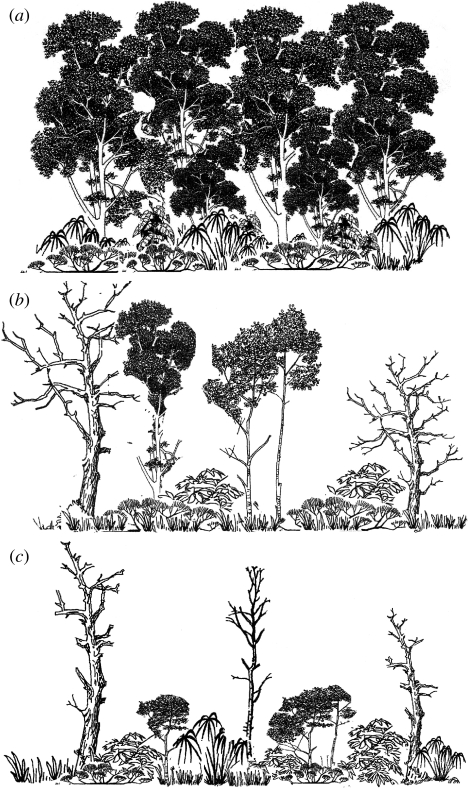
Vertical cross-sectional profiles representing the sequential ‘secondarization’ of repeatedly burned forests in central Amazonia. This trajectory departs from (*a*) an unburned closed-canopy forest to (*b*) a once-burned open-canopy forest where nearly half of the canopy trees had been either damaged or killed, to (*c*) a twice- or thrice-burned forest that resembles young secondary forests growing on degraded land. This forest secondarization process involves a number of drastic changes in forest structure and composition, including severe loss of biomass, loss of vertical structure resulting from upper canopy thinning and the loss of emergent trees, hyper-proliferation of pioneers, rapid compositional turnover and seed-bank simplification (see text).

Data presented in this study provide novel insights into the ecological consequences of repeated fire disturbance in Amazonian seasonally dry forests. Our principal observation is that repeated pulses of fire-induced tree mortality lead to a rapid collapse in the abundance of old-growth trees, and a community turnover that is analogous to the ‘secondarization’ of primary forests (figures 2 and 3), with significant shifts in forest composition occurring with each consecutive fire event. This fire-mediated cascade in the species composition of Amazonian forests is underpinned by five interrelated processes.

- *Loss of primary forest species*. Many studies have documented the intrinsic vulnerability of primary forest trees to fire stress (e.g. Uhl & Kauffman 1990; Pinard & Huffman 1997; Cochrane & Schulze 1999; Barlow *et al*. 2003*a*,*b*). Fires may promote the secondarization or impoverishment of large areas of forests by acting as local extinction filters, extirpating species that are unable to tolerate thermal stress induced by fire contact. The consequences of this sudden selective pressure in core Amazonian forests are evident by the generally lower rates of fire-mediated tree mortality along the phytogeographic fringes of the Amazon (see Barlow & Peres 2006*a*), where forests close to savannah ecosystems have been more frequently exposed to fire disturbance.
- *Changes in forest regeneration patterns*. Our results suggest that episodic patterns of post-burn tree recruitment are strongly influenced by the number of times a forest had burned, with a different suite of pioneer species dominating the vegetation composition after each fire event (table 1). This may be due to the fire-induced destruction of the residual seed bank, mortality of entire cohorts of pioneer species, post-burn availability of diaspore sources, seed and seedling predation, soil infertility and competition with grasses and ruderal species, all of which can alter the rates of forest regrowth and biomass accumulation, and, under extreme conditions, deflect or arrest successional processes. Although these patterns could also be explained by a stochastic founder effect, with the most abundant species being those that colonized first after each recurrent fire, this alternative explanation is less likely as it fails to explain the almost complete absence of long-lived pioneers in areas that have undergone recurrent fires.
- *Susceptibility of pioneer species to fire*. The pulse of plant recruitment dominated by long-lived pioneers following low-intensity surface fires provides a dense regrowth that may partly compensate for the loss of primary forest tree and liana species, even though the composition and structure of the forest remains very different from unburned forests (figures 1*b* and 2*b*). However, these long-lived pioneer species are themselves highly vulnerable to any subsequent fire (which in any case tends to consume more fuel and is more intensive). For example, pioneers that were common in once-burned forests (such as *J. copaia* and *Cecropia* spp.) were much less abundant or entirely absent in forests that had succumbed to recurrent fires (table 1). These species also suffer some of the highest mortality rates (compared with any tree species) following low-intensity understorey fires in other Amazonian primary forests (Pinard *et al*. 1999; Ivanauskas *et al*. 2003). The enhanced susceptibility of the dominant tree species regenerating after a single low-intensity fire event increases the overall plant mortality rate in forests that burn more intensively for a second time, thus reinforcing the positive feedback cycle in which recurrent fires erode the vegetation structure of previously unburned forests (cf. Cochrane *et al*. 1999; Nepstad *et al*. 1999).
- *Vulnerability of resprouting stems to fire*. The ability to resprout following disturbance provides many trees with a mechanism to survive fire events, and these regenerating stems play a crucial role in fostering a species-rich regeneration (Guariguata & Ostertag 2001). However, these resprouting stems are themselves highly vulnerable to subsequent fire stress (Uhl *et al*. 1981), further exacerbating the process of impoverishment and secondarization of tropical primary forests that undergo more than one fire event.
- *Changes in the availability of seed dispersal agents and seed shadows*. In addition to their direct effects on forest structure and composition, fires are likely to play a more subtle long-term role in determining the future forest dynamics and composition, as recurrent fires also have a devastating effect on animal populations, including many of the large-bodied vertebrates that disperse large-seeded species (Peres *et al*. 2003; Barlow & Peres 2004, 2006*b*). Local extinctions and population declines in mid-sized to large-bodied frugivores—resulting from both the short-term mortality in the aftermath of fires and the long-term changes in habitat quality (e.g. structure and food supplies)—have both qualitative and quantitative consequences to patterns of seed dispersal in repeatedly burned forests. These are likely to aggravate dispersal limitation of propagules in many large-seeded endozoochorous plants, while favouring small-seeded and wind-dispersed plants (Stoner *et al*. 2007; Wright *et al*. 2007). This is essentially analogous to changes in animal-mediated seed dispersal in comparatively better-understood scenarios of forests that were semi-defaunated by overhunting, where the availability of seed vectors is depressed without major changes in habitat structure (Peres & Palacios 2007).

## 5. Patterns of tree mortality

Our data from a small number of forest plots support other tropical forest studies that demonstrate high rates of post-fire tree mortality (See Barlow & Peres (2006*a*) for a summary of mortality from 14 studies), and provide further evidence that tree mortality may be highest in both the smallest and largest size classes (see also Williams *et al*. 1999; Barlow *et al*. 2003*b*). We do not attempt to estimate the loss of forest biomass (and committed carbon emissions) in this study, as accurate estimates would require information on (i) soil carbon pools, (ii) non-lethal tree damage, (iii) lowered biomass of senescing trees (Chambers *et al*. 2001), and (iv) wood density of different species (particularly regenerating pioneers). However, these data clearly demonstrate that forest fires have the potential to offset any potential carbon sink from the Amazon forest (cf. Barlow & Peres 2004) and further contribute to increasing global carbon emissions (Lewis 2006).

## 6. Evaluating the savannization paradigm

Various terms have been used to describe the predicted ‘dieback’ of Amazonian forests. We recorded five such terms stated by climatologists and forest ecologists at the fate of the Amazon conference in Oxford (March 2007; this issue), including ‘savannization’, ‘pasteurization’, ‘desertification’, ‘secondarization’ and ‘scrubification’. Different terms are inevitable where there is regional variability in the nature of threats, and different authors have slightly different world views on the nature of the process involved (cf. Verstraete 1986). However, we argue that some of the current terms describing climate-related ecosystem transitions (such as savannization and desertification) are misleading and technically inaccurate because their outcomes are driven solely by the direct effects of climate change on vegetation and they fail to consider indirect mortality rates (mediated through fire).

It is our view that any increase in dry season length brought about through climate change is highly likely to be accompanied by widespread increases in forest flammability (Nepstad *et al*. 2004) and subsequent large-scale fire events (e.g. Cochrane 2003; Aragão *et al*. 2008). It is well established that these fires bring about severe compositional and structural changes in Amazonian forests (e.g. this study; Cochrane & Schulze 1998, 1999; Barlow & Peres 2004). These changes are likely to be significantly more severe than changes in forest structure and composition that could occur through drought stress under moderate scenarios of climate change, especially considering that Amazonian forests appear to be more resilient to drought stress than previously thought (Nepstad *et al*. 2007; Saleska *et al*. 2007).

If we accept that fire will be an integral part of climate-driven ecosystem change, then how would Amazonian forests look in a hotter, drier future? Our data provide a unique insight and suggest that recurrent fires can lead to an ecosystem phase shift from pristine closed-canopy primary forests to more open forests dominated by short-lived pioneer species, which reflects patterns observed in humid tropical forests disturbed by logging, fires and edge effects elsewhere (Slik *et al*. 2002; Santos *et al*. 2007). In particular, these forests are not dominated by woody species typical of natural neotropical savannahs (as implied by the savannization paradigm), but are more similar to young secondary forests regenerating on degraded lands. The changes brought about by fire involve a number of drastic alterations in forest structure and composition that clearly mimics the ephemeral formation of young second-growth stands, including a severe collapse in forest phytomass, loss of vertical structure resulting from severe upper canopy thinning and the loss of emergent trees, hyper-proliferation of pioneers, rapid compositional turnover and seed-bank simplification (figure 3). This structural collapse is accompanied by a severe functional impoverishment in the life-history traits of residual stands with profound consequences for ecosystem services such as carbon retention and water cycling. For example, the high diversity of slow-growing shade-tolerant trees typical of unburned, old-growth forests (e.g. large-seeded, emergent and hardwood tree species) is rapidly replaced by a small dominant set of persistent, fast-growing pioneer species that accrue a low biomass due to their smaller size and low wood density.

## 7. Conclusion

This paper outlines the long-term consequences of forest fires in one region of the Brazilian Amazon, and shows how fires drastically alter forest structure and composition, leading to a rapid impoverishment of even previously intact primary forests over a relatively short period of time. Such fires are likely to have long-term impacts on tree species composition even if recurrent fires can be prevented through effective fire suppression practices. For example, many modern tropical forests show evidence of catastrophic disturbance events (Baker *et al*. 2005; Pitman *et al*. 2005), and compositional recovery from forest disturbance, when observed over longer time scales, is always slow and of the order of hundreds of years, if not millennia (Turner *et al*. 1997; Charles-Dominique *et al*. 2003; Pitman *et al*. 2005).

These results clearly highlight the importance of preventing fires from spreading further into new Amazonian forest frontiers. Although biomass burning is widely practised by virtually all rural peoples in the Amazon, it is also one of the few aspects of climate change mitigation over which we retain some direct control. Some small-scale agricultural projects have been highly successful in reducing the use of fire in Amazonian smallholdings (e.g. see Silva *et al*. (2006) and the project ‘Roça sem queimar’). Expanding and developing these projects to reduce the availability of ignition sources should be an urgent conservation priority over the coming decades.

## Figures and Tables

**Table 1:** Tree species and genera from the 10–20 cm DBH size class (and shrubs and saplings below 10 cm in DBH) which were most abundant in each burn treatment, showing a high degree of turnover in community composition with each additional burn. (All species (or genera) with a density greater than 10 trees ha^−1^ are shown for trees 10 cm and above in DBH, and the most abundant species in once-, twice- and thrice-burned forest plots are shown for saplings.)

species	family	forest type where most abundant	trees (10–20 cm in DBH) ha^−1^			

			unburned	once-burned	twice-burned	thrice-burned
*Protium and Tetragastris* spp.	Burseraceae	unburned	69	15	2	2
*Pouteria* and others	Sapotaceae	unburned	17	13	0	0
*Sclerolobium and Tachigali* spp.	Fabaceae	unburned	17	4	0	0
*Rinorea* spp.	Violaceae	unburned	14	0	0	0
various genera	Lauraceae	unburned	12	2	4	0
*Cecropia* spp.	Cecropiaceae	once-burned	0	69	22	8
*Jacaranda copaia*	Bignoniaceae	once-burned	0	18	0	0
*Pseudobombax* sp.	Malvaceae	twice-burned	0	0	88	14
*Inga* spp.	Fabaceae	twice-burned	8	0	22	10
*Tapirira* sp.	Anacardiaceae	twice-burned	0	0	14	0
*Cordia* sp.	Boraginaceae	thrice-burned	1	2	0	30
			saplings (<10 cm in DBH) per 200 m^2^			
*Palicourea guianensis*	Rubiaceae	once-burned	—	38	0	5
*Aparisthmium cordatum*	Euphorbiaceae	twice-burned	—	13	79	12
*Cordia* sp.	Boraginaceae	thrice-burned	—	4	5	30
